# Low-loss ultrafast and nonvolatile all-optical switch enabled by all-dielectric phase change materials

**DOI:** 10.1016/j.isci.2022.104375

**Published:** 2022-05-07

**Authors:** Qiang He, Zhiyuan Liu, Yitao Lu, Guoxun Ban, Hao Tong, Yi Wang, Xiangshui Miao

**Affiliations:** 1 Wuhan National Research Center for Optoelectronics, School of Integrated Circuits and School of Optical and Electronic Information, Huazhong University of Science and Technology, 1037 Luoyu Road, Wuhan 430074, China; 2 Hubei Yangtze Memory Laboratories, Wuhan 430205, China

**Keywords:** Photonics, Applied sciences, Materials science

## Abstract

All-optical switches show great potential to overcome the speed and power consumption limitations of electrical switching. Owing to its nonvolatile and superb cycle abilities, phase-change materials enabled all-optical switch (PC-AOS) is attracting much attention. However, realizing low-loss and ultrafast switching remains a challenge, because previous PC-AOS are mostly based on plasmonic metamaterials. The high thermal conductance of metallic materials disturbs the thermal accumulation for phase transition, and eventually decreases the switching speed to tens of nanoseconds. Here, we demonstrate an ultrafast switching (4.5 ps) and low-loss (2.8 dB) all-optical switch based on all-dielectric structure consisting of Ge_2_Sb_2_Te_5_ and photonic crystals. Its switching speed is approximately ten thousand times faster than the plasmonic one. A 5.4 dB on-off ratio at 1550 nm has been experimentally achieved. We believe that the proposed all-dielectric optical switch will accelerate the progress of ultrafast and energy-efficient photonic devices and systems.

## Highlights

• All-dielectric phase change materials are used to achieve low loss all optical switch
• Only 15 nm phase change film is used for laser induced ultrafast switching
• Up to 7.4 dB switching contrast can be realized in the Near Infrared Spectrum
• Nano-hole array metasurface enables polarization insensitive optical filtering

## Graphical abstract

**Figure:**
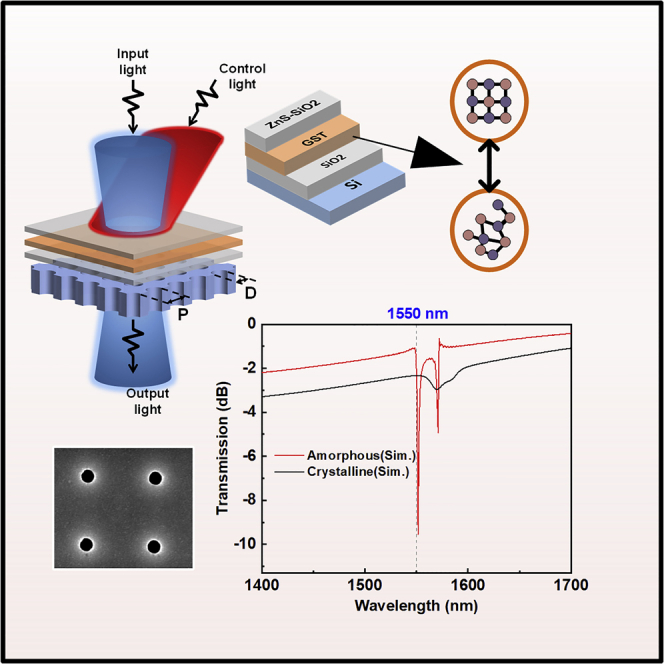

## Introduction

Because the research of silicon-on-insulator (SOI) components has yielded a number of exciting results (Idjadi and Aflatouni, 2017)(Nielsen et al., 2016)(Zhao et al., 2018), optical systems on chips are attracting more and more attention. All-optical systems are different from the electrical ones, and no major electrical components are involved in these systems because the bottle neck inside the electronic device slows down the speed of data transmission (Gholipour et al., 2013)(Stern et al., 2014). For all-optical systems, not only the communication system but the optical brain (e.g., all-optical CPU) needs efficient, stable and long lifetime switch devices between data nodes. As one of the most fundamental devices, all-optical switches with quite different mechanisms have been reported for many times. For instance, light could be manipulated by tuning carbon nanotubes (Chen et al., 2002) to control plasmonic metamaterials, or using Mach-Zehnder interferometer mechanism to realize optical switching, or tuning ring resonator with chalcogenide compounds to control optical signal (Heebner et al., 2004)(Stegmaier et al., 2017). Despite this progress, a more reliable and faster switch device with a simpler structure and lower energy loss is yet to be developed.

Phase-change all-optical switches based on chalcogenide materials attract massive attention for its non-volatile property and cycling ability. It is always a structure of chalcogenide compounds coupling with plasmonic metamaterials (Gholipour et al., 2013)(chen et al., 2013), or chalcogenide compounds metasurface (Choi et al., 2019)(Zhu et al., 2021). Basically, chalcogenide compound materials are widely exploited in non-volatile optical and electronic data storage, and in recent years in displays as well (Wełnic and Wuttig, 2008)(Wuttig et al., 2017)(Hemmatyar et al., 2021). As the most typical Chalcogenide compound materials, Ge_2_Sb_2_Te_5_ (GST) can be reversibly switched between two stable states, the amorphous state and the crystalline one. Different from most solids in which the amorphous and crystalline states have very similar optical property, phase change materials present a pronounced contrast of refractive index and extinction coefficient (Kohara et al., 2006)(Feinleib et al., 1972)(Akola and Jones, 2007)(He et al., 2020). The reversible phase transition can be induced by either programmed laser pulses or electric pulses. It is reported that GST can be switched for 10^12^ times between two states, and can store the information for nearly 10 years in room temperature (Nardone et al., 2012)(Lu et al., 2013)(Kim et al., 2016).

Because of surface plasmon resonance, the artificial metal arrays, which show surface frequency-selectivity property and perfect absorbance, have been investigated for light tuning and color printing(Shu et al., 2014)(Ahmadivand et al., 2017)(Tittl et al., 2015)(Tian and Li, 2016)(Kumar et al., 2012). However, because it is based on metallic metamaterials, high loss of metal increases the device loss and high conductance of metal weakens the thermal accumulating for the transition process, which may have negative effect on the device switching speed (Kuznetsov et al., 2016). Recently, the tunable all-dielectric metasurface has been explored (Staude et al., 2018)(Sautter et al., 2015) and used in all-optical switches (Zhang et al., 2019). However, most of the previous studies are based on nano-grating structure, which is sensitive to polarization (Karvounis et al., 2016)(Gholipour et al., 2018).

In this work, we proposed a novel all-optical switch, which combines all the aforementioned approaches. It is claimed that all-dielectric metamaterials have lower losses, which means that the power consumption can be remarkably decreased if we make them into switches (Boltasseva and Atwater, 2011). Dielectric materials can also decrease the thermal loss because of its lower thermal conductance. Hence, an all-optical switch based on chalcogenide phase-change materials and photonic crystals (PhCs) with ultrahigh switching speed and low loss has been investigated. It is beneficial for improving switching performance for PC-AOS. We first present our theoretical design for our device concept. Then, we give a typical example of the fabricated optical switch at 1550 nm wavelength. Finally, we describe our demonstration of all-optical reversible switching in the femtosecond regime.

## Results and discussion

### Proposed phase change all-optical switch

**Figure 1:**
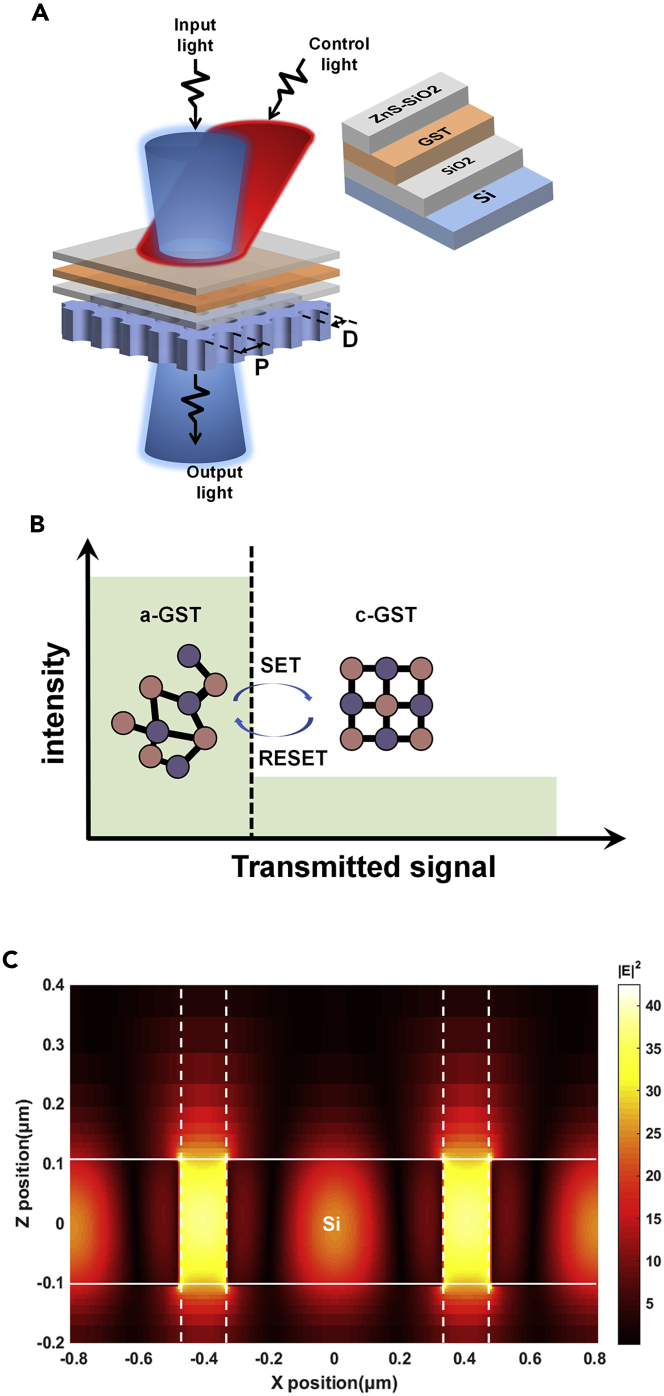
Operation principle of the proposed phase change all-optical switch (A) The phase change materials thin-film is sandwiched by ZnS/SiO_2_ and SiO_2_. Laser pulses control the phase transition between amorphous and crystalline states, which show dramatic refractive index difference. The resonance wavelength can be finely tuned by changing diameter D and periods P. (B) The change of transmitted light intensity between amorphous and crystalline states, which are respectively schematic diagrams of the atomic arrangements. (C) The X-Z plane electric field power distribution at the resonance frequency in a single Si layer with submicron holes structure. The period of holes p = 810 nm, the diameter of holes D = 150 nm, the thickness of Si layer is 210 nm.

Our design of the PhCs on silicon substrate is sketched in Figure 1A. It consists of a thin-film of PhCs, a 25 nm SiO_2_ film, a 15 nm GST film and a 100 nm ZnS/SiO_2_ film. Phase change materials (GST-225 in this work) act as the dynamic component, which is sandwiched on silicon PhCs. There are dramatically large differences in optical constant between the long-range disorder amorphous phase and long-range order crystalline phase. To control the reversible phase-change processes we formed trains of optical pulses of different duration, typically consisting of a few tens’ femtosecond laser pulses. It manifests as changes in transmittance intensity and shifts in the spectrum (Figure 1B). PhCs have already been realized to limit the electromagnetic wave inside the optical bandgap (Fano, 1961). As shown in Figure 1C, the electric field at the resonance frequency is localized in a single Si layer with PhCs structure. This phenomenon called Fano resonance is because of the interactions of narrow Bragg resonances with broad Mie or Fabry–Pérot bands in photonic crystals (Luk’yanchuk et al., 2010). Fano resonance can provide a quite steep and asymmetric transmission spectrum (Hayashi et al., 2016)(Yu et al., 2016), which is beneficial for some sensing devices. Similar to hybrid plasmonic metamaterials (Wang et al., 2015)(Nikolaenko et al., 2010), the size parameters and the refractive index (or dielectric constant) of PhCs surface can influence the resonance frequency (Petronijevic and Sibilia, 2016). Thus, by tuning the structure dimension and surface refractive index, the spectrum of the device may red- or blue-shift so that the selection of the switching wavelength and the switching function can be finely realized.

**Figure 2:**
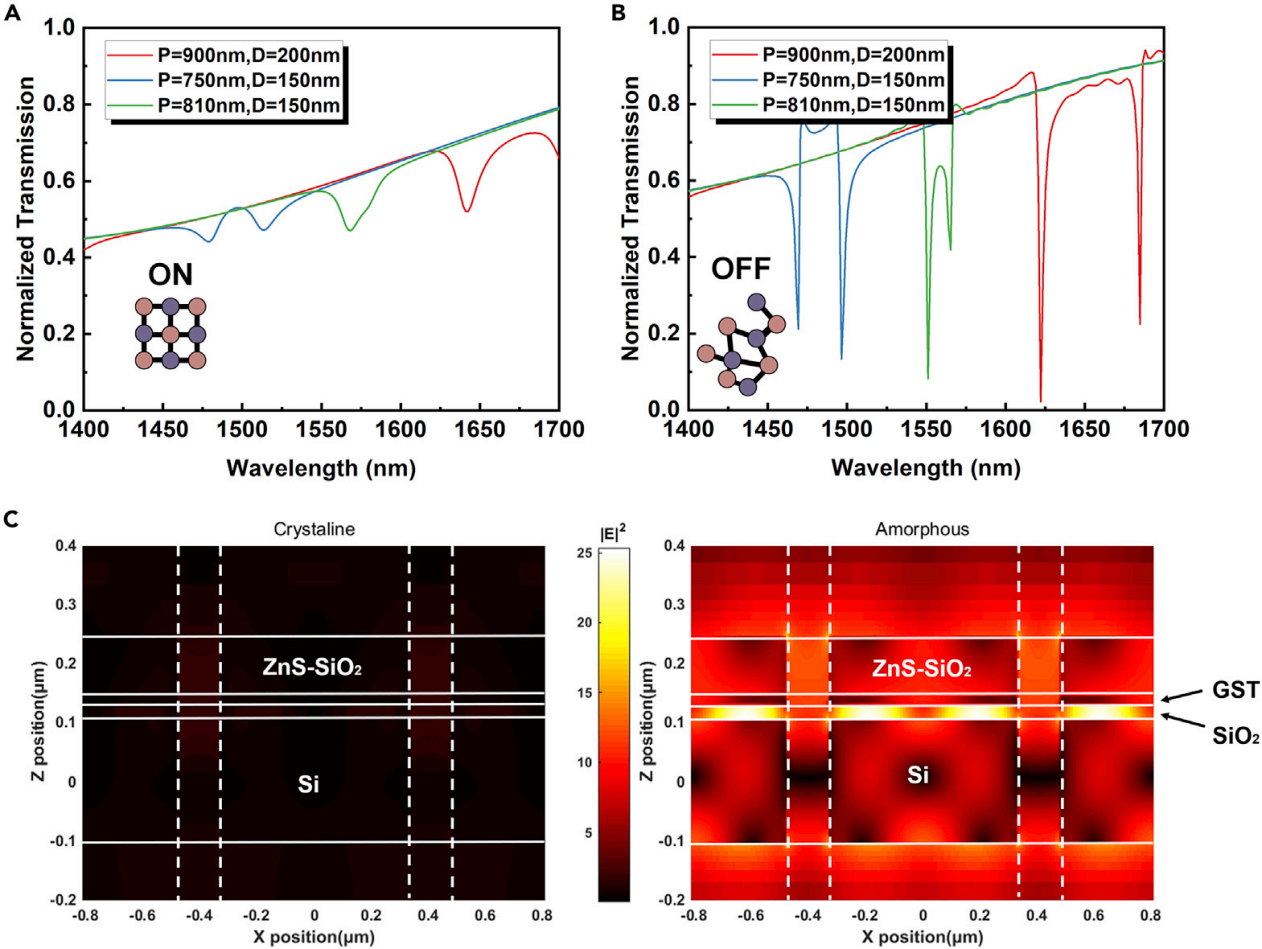
Transmission and electric field simulations (A and B) The transmission spectra under holes with different size parameters when GST is (A) crystalline and (B) amorphous respectively. Where P represents the holes’ period and D represents the holes’ diameter. (C) The X-Z plane electric field power distribution at the resonance frequency of proposed switch film. Most electric field power is limited inside the film’s range.

Figures 2A and 2B compare the calculated transmission between crystalline and amorphous states, respectively, when varying the period P and diameter D. This plot shows obvious transmission contrast between the two states. In the crystalline state, most light transmits through the film, thus we call this ON state. By contrast, in the OFF state, the normalized transmission spectra are sharp and quite lower when the GST is amorphous. In addition, the frequency selectivity of the transmitted light means that this PC-AOS can provide a wide tunability across the near infrared spectrum. From the perspective of Fano resonance, the holes-induced eigenmodes and quasi-continuous cavity mode play the roles of the narrow resonance and broad resonance, respectively. The Fano line shape is the result of interference between them. The electric field distributions of crystalline and amorphous states are simulated by the Lumerical Finite Difference Time Domain (FDTD) Solution, as shown in Figure 2C. Apparently, in the spacing layer between the GST and Si, there exists strong electric field confinement owing to Fano resonance when GST is amorphous. And the field enhancement effect occurs at the resonance wavelength, which is slight at crystalline state.

### Device image and switching characteristics

**Figure 3:**
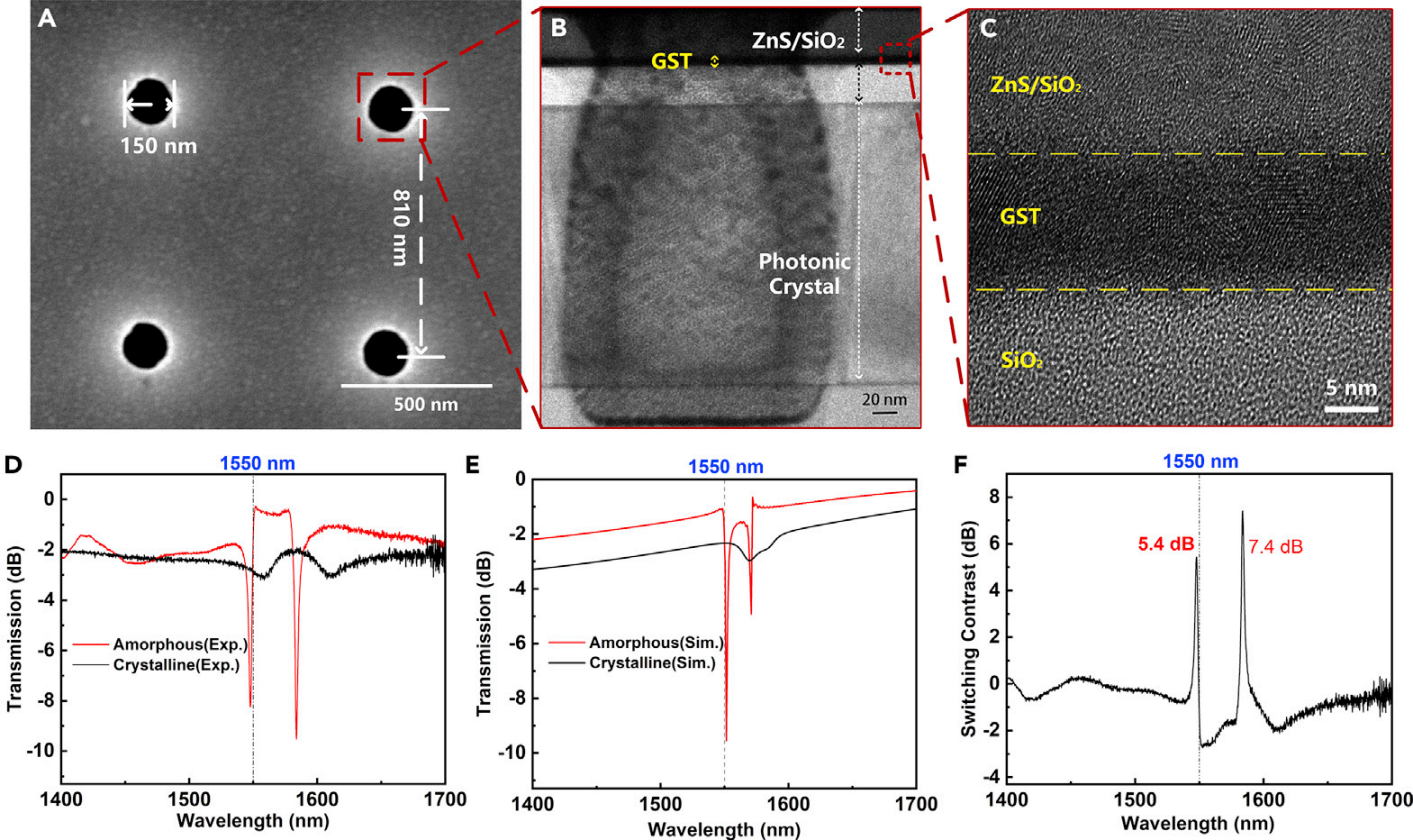
Image and optical properties of the proposed PC-AOS device (A) A SEM image of PhCs. The diameter of each submicron hole is 150 nm, and the distance between them is approximately 810 nm. (B) A TEM image of the cross section of the device. The boundary of films can be clearly observed. The layer of ZnS/SiO_2_ is thinner because of the ion beam irradiation. (C) TEM image of ZnS/SiO_2_-GST-SiO_2_ sandwich films. The as-deposited state of GST is crystalline and no amorphous region is observed. (D) Transmission spectra of the device when the GST is amorphous state (red line) and crystalline state (black line). To compare the figure, we carry on a logarithm analysis. Two sharp valleys can be seen around 1550 nm in amorphous state and they shift to larger wavelengths in crystalline GST. (E) Simulation results of two GST phases. (F) Switching contrast of two states. Amorphous state is set OFF state and crystalline state is set ON state. The difference between them is caused by the actual refractive index. Two peaks are available. The contrast is about 5.4 dB for optical communication wavebands.

We next design and experimentally demonstrate a phase change optical switch at 1550 nm wavelength. After electronic beam exposure, we etched the silicon substrate filmed with ZEP520 photoresist for sub-micron holes by inductively coupled plasmon (ICP) technique. From the scanning electron microscope (SEM) image in Figure 3A, the diameter of the submicron holes is 150 nm, and the distance between each two-unit cell is 810 nm. The transmission electron microscope (TEM) image of the entire device in Figure 3B confirms that the depth of the submicron holes is approximately 210 nm. GST is amorphous when it is deposited with magnetron-sputtering technique. The TEM image of the first three layers is shown in Figure 3C. The atoms’ lattice symmetry can be found in the GST layer. The device in a square area of 81 × 81 μm^2^ has 10,000 (100 by 100 array) submicron holes in total.

To improve the extinction contrast of the device, an extremely thin SiO_2_ film beyond PhCs was deposited to lower the environmental dielectric properties and then an amorphous GST layer of 15 nm was sputtered above. On top of the GST film, there was a capping layer to isolate GST from air, otherwise it would be easily evaporated or oxidized when we perform phase transition with laser pulse or annealing. We choose ZnS/SiO_2_ which is oxidation-proof as the capping layer. This capping layer can also decrease the possibility of material loss (Wang et al., 2016)(Li et al., 2016).

To identify the direction of phase transition, we first annealed the sample to get a complete crystalline state and then focused on the re-amorphization. The phase transition of GST is a thermodynamic process. The crystallization temperature (*T*_c_) and melting temperature (*T*_m_) of GST are reported to be 160°C and 600°C (Terao et al., 2009)(He et al., 2014). To achieve full crystallization especially in a large area, the heating process takes a relatively long time and the temperature has to be kept beyond *T*_c_ and below *T*_m_. It is, however, much easier for a small area to change its phase by laser pulse stimulation. A complete crystallization phase transition can be induced by a series of laser pulses or single pulses with particular energy and time duration (Michel et al., 2014)(Behrens et al., 2019). In addition, dielectric materials are different from metallic materials in thermal accumulation. The process from crystalline state to amorphous state requires higher energy. An all-dielectric structure may have a larger influence on this process.

Because the as-deposited GST is amorphous, the sample is annealed in a furnace at 250°C for an hour. The transmission is characterized using a near-infrared spectrum microscope, as shown in Figure 3D. When GST is amorphous, a deep and sharp valley near 1550 nm is observed. After annealing, the valley has red-shifted, and the depth is shallower than the former because the refractive index increases when GST turns into the crystalline state. The loss of the device is measured as 2.8 dB, which is lower than the loss of switching based on plasmonic metamaterials (Gholipour et al., 2013)(Kang et al., 2021). The simulation results are shown in Figure 3E. It is slightly different from the measurement (such as transmittance, peak position), which is because of the size vibration and the optical constant differences of the material between fabrication and simulation. The switching contrast is calculated to be 5.4 dB near 1550 nm by comparing the ON-state and the OFF-state spectra from Figure 3D. The contrast is higher than other similar works, and an even higher contrast can be obtained at 1583 nm which is 7.4 dB, as seen in Figure 3F.

### Reversible optical switching with femtosecond laser

We next performed a femtosecond laser induced re-amorphization for the PC-AOS devices. Phase transformation from amorphous to crystalline state can be achieved by both annealing and a wide laser pulse with particular power. In comparison, the re-amorphization (from crystalline state to amorphous state) of GST is quite demanding, because the crystal lattice needs to be molten and rapidly cooled to room temperature to avoid crystallization. Hence, a narrow pulse with higher power can switch it back to glass (Boltasseva and Atwater, 2011). However, when the pulse width is only femtosecond or sub-picosecond scale, the thermal energy is easily dissipated, as a consequence, the temperature is not the only reason that is responsible for the phase transition (Hu et al., 2015)(Huang et al., 2011).

**Figure 4:**
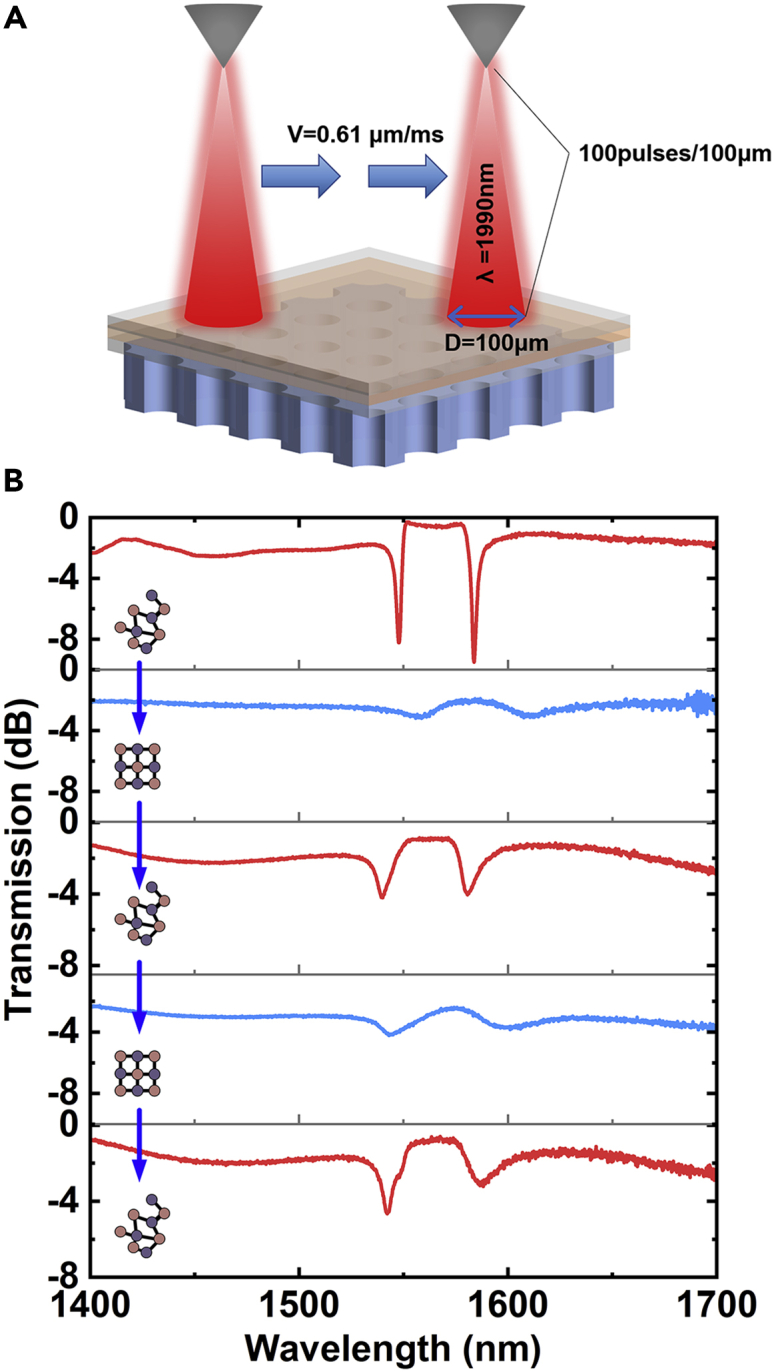
Schematic diagram and spectra of the laser pulse-induced reversible process (A) The probe moves with a speed of 0.61 μm/ms in one direction and scans over a line area across the PhCs. In this area, about 100 pulses are applied to the film in every 100 microns. (B) The transmission spectrum of two ON/OFF cycles. The positions of resonance of all the three amorphous states are exactly the same. The spectra undergo a blue-shift when the GST goes to crystalline state.

To re-amorphize the GST, a sequence of femtosecond laser pulses is generated as the “control light”. But if the pulse energy is accumulated in a small area, the localized heat could rise quickly, and the internal stress may destroy the capping layer. To avoid this, we controlled the pulse number by shifting the laser probe. We set the laser energy density to 35 mJ/cm^3^, and the pulse band is 45 fs with the repetition frequency of 1 kHz. The wavelength of the laser beam is 1990 nm. Reducing the number of pulses applied on the phase change material was achieved by moving a femtosecond laser rapidly. Because the light spot diameter is around 100 μm, the speed of the pump probe moving is 0.61 μm/ms on average (the method is described in Figure 4A). Approximately 100 pulses are applied to the device within a distance of 100 μm. Considering there is a path of acceleration, the beginning is offset by several millimeters away from the testing region. The light trace can be observed with human eyes after laser exposure.

We then realize the cycling characteristics of the switch using the aforementioned method. Two ON/OFF cycles are shown in Figure 4B. It started as the same as Figure 3D where GST was as-fabricated amorphous. Then the crystalline state of GST was obtained after annealing. By moving the laser probe, the pulse-induced re-amorphous GST results in a spectrum shifting that is similar to the initial state. Then we repeated this cycle again. The spectrum turns back to the former. The switching time was determined as around 4.5 ps.

**Figure 5:**
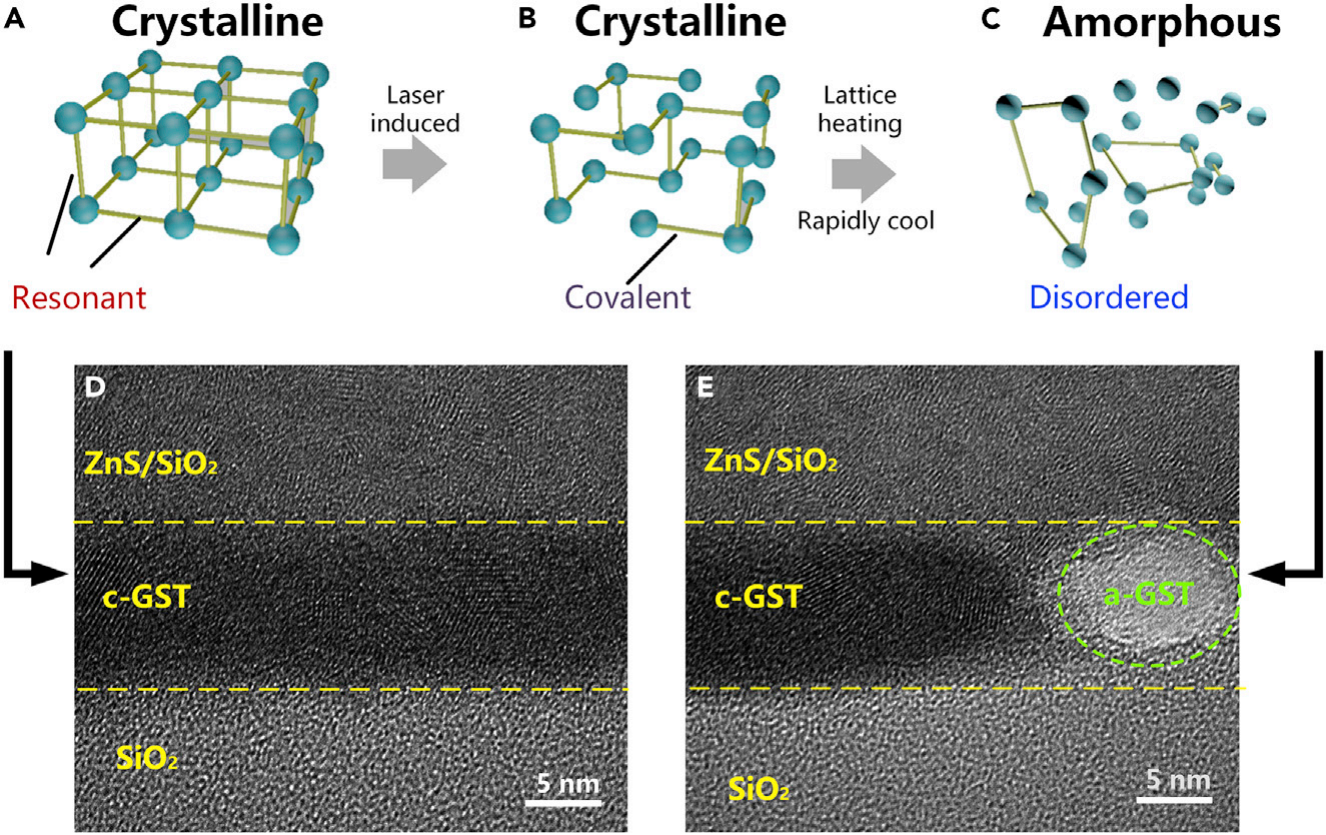
Atomic structure and TEM observation of a- and c-GST (A–C) The schematic diagram of ultrafast laser treatment in GST. Resonant bonds prevail in the crystalline state. The ultrafast laser can break the bonds without collapsing the crystal lattice within picoseconds transiently. However, the residue heat from the laser will finally break the structure, making some GST turns into an amorphous state. (D) The TEM image of crystalline state. The yellow dashed lines are borders between layers. The GST film shows apparent lattice symmetry. (E) The TEM image of laser-induced GST state after process a to c. Partial amorphization can be seen in the green dashed circle (c-GST and a-GST denote crystalline and amorphous GSTs in the figures).

The change of optical properties in chalcogenide materials is usually caused by structure transition (Hosokawa et al., 2012). The atoms have been re-aligned from amorphous to crystal structure, whereas the reversed process requires high energy to get a melt-quenched amorphous state. However, in the picosecond timescale pulse-induced phase change, it is considered that the atoms have no time to recombine. According to recent research, the large difference in dielectric function between amorphous and crystalline GST is caused by resonant bonds in the crystalline state (Shportko et al., 2008)(Huang and Robertson, 2010). It is reported that femtosecond optical excitation can instantaneously break the resonant bonds in crystalline state (Waldecker et al., 2015), leading to a drop of dielectric function when the phase is changed. As more of such bonds are depopulated, the larger percentage change of dielectric function can be achieved (Shportko et al., 2008). When the sub-picosecond or femtosecond laser pulses are appealed to GST, the resonant bonds are broken and the optical property changes, such as the re-amorphization process in Figure 4B. The laser power not only breaks the resonant bonds instantaneously but changes the optical properties before ionic motion has occurred. The remaining energy could also heat the lattice, which thermally melts the long-range order after several picoseconds (Waldecker et al., 2015). So that GST can be re-amorphized in picoseconds. We indicate this mechanism in Figure 5. Because of the rapid heating, it is very probable that the laser re-amorphizes the materials without melting (Kolobov et al., 2011)(Li et al., 2011)(Nam et al., 2012). Figure 5D is the TEM image after annealing and represents the crystalline GST as in Figure 5A. The crystalline lattices are arranged orderly in the GST layer. After the laser exposure, the TEM image shows a mixture of amorphous and crystalline phases in Figure 5E. This is because of the heating by the residue energy of laser pulses after breaking the resonant bonds (Figure 5C).

Regarding the picosecond-level switching speed shown by the structure proposed in this paper, we have considered a reasonable interpretation of this result from the perspective of non-thermal effects in (Waldecker et al., 2015). Artificial metal arrays will extract the energy used to depopulate the resonant bonds before the lattice heats above the *T*_m_. Energy extraction could be realized in nanostructured devices by rapid transfer of the photoexcited carriers, both electrons and holes, into a metal or semimetal, and resonant bonds could re-establish and recover the crystalline-state optical properties on the few-picosecond timescale. We believe that the all-dielectric structure inhibits the recovery process of the resonant bond, so a faster switching speed has been achieved than the metallic metamaterial all-optical switch.

Then we compare the proposed PC-AOS with other existing works. Table 1 summarizes various optical switch designs based on phase change materials. The proposed all-dielectric PC-AOS achieves 2.8 dB loss which is lower than most plasmonic all-optical switches. The switching contrast is 5.4 dB for a 1550 nm laser, which is more than enough for optical communication device applications. In addition, the thickness of the GST active layer used in the optical switch designed in this paper is thinner, which means that the phase change process can be achieved by using a laser with a lower energy level. In addition, the proposed all-dielectric PC-AOS can be switched at picosecond timescale, which is comparable with the other all-optical switch systems, such as Mach-Zehnder based and Kerr effect based ones. A thinner GST film also enhances the switching contrast of this device. The SiO_2_ film between GST and PhCs can decrease the average surface refractive index so that the switching contrast of the device can be further increased.

**Table 1:** Comparison of proposed optical switches with existing designs

Ref	Method	Switching Ratio	Switching speed	Insertion Loss	Remark
Gholipour et al. (2013)	PCM/Au metasurface	4 dB	200 ps	3 dB	Optically driven
Chen et al. (2013)	PCM/Au metasurface	6.5 dB at 1890 nm	NA	1.4 dB	Optically driven
Choi et al. (2019)	GST metasurface	7 dB	NA	NA	Optically driven
Michel et al. (2014)	Au/VO_2_ metasurfaces	13.7 dB at 2200 nm	100 fs	3.3 dB	Volatile
Zhang et al. (2021)	PCM/Au metasurface	10.4 dB at 1640 nm	NA	0.82 dB	Electrically driven
Abdollahramezani et al. (2021)	GST metasurface	6.6 dB at 1430 nm	NA	3.0 dB	Electrically driven
This Work	PCM/All-dielectric photonic crystal	5.4 dB at 1550 nm 7.4 dB at 1580 nm	4.5 ps	2.8 dB	

## Conclusion

In summary, an freespace all-optical switch has been demonstrated by incorporating the phase change material with low loss all-dielectric metamaterials. By altering the structural parameters, the PhCs show the switching property in different wavebands. We have confirmed that the switching function can be realized by femtosecond laser. The proposed PC-AOS features a multi-film coated structure based on SOI substrate, which adapts to modern CMOS integrated technique. Thus it enables massive manufacture possible. Integrated with optical waveguide, the device can be more practical for all-optical communication. The signal transmission of “node to node mode” in optical communication can be safer and more efficient. Theoretically, the cycling of GST film can achieve trillions of times, which can be superior to some other similar devices. This work also provides a platform for researching the physical mechanism of metavalence.

### Limitations of the study

In this study, we have simulated, fabricated and characterized GST-225 based all optical switch, which can be working at freespace. The switching property can occur at various wavebands by fine-tuning the periods and diameters of nanoholes. In this design, we use a periodically arranged dielectric nano-hole array, which is symmetrical. Thus, the proposed device does not depend on the polarization of the signal light. In addition, we use only 15 nm GST as the active layer, which is more favorable for light control. Using all-dielectric materials enables the PC-AOS, thus showing a lower loss than other existing designs. In addition, we carried out laser induced switching experiments for investigating the reversible ON/OFF property. However, only several cycles have been performed currently; one could perform a longtime endurance test to demonstrate the cycling ability.

## STAR★Methods

### Key resources table

**Table:** 

REAGENT or RESOURCE	SOURCE	IDENTIFIER
**Software and algorithms**		

FDTD	Lumerical Co	https://www.lumerical.com/products/fdtd/

**Other**		

ICP Etching	Oxford Plasmalab system	N/A
PlasmaPro 800 PECVD	Oxford Instruments	https://plasma.oxinst.com/products/pecvd/plasmapro-800-pecvd

### Resource availability

#### Lead contact

Further information and requests for resources and reagents should be directed to and will be fulfilled by the lead contact, Dr. Hao Tong (tonghao@hust.edu.cn).

#### Materials availability

This study did not generate new unique reagents.

### Experimental model and subject details

The three-dimensional finite-difference time-domain (FDTD) method has been employed to analyze the E-field characteristics of the proposed PC-AOS devices. In these numerical simulations, the propagation direction of incident wave is set to be perpendicular to the x-y plane where the phase change all-dielectric metamaterial pattern array lies. The refractive index of amorphous and crystalline GST are 3.3 + 0.01i and 4.3 + 0.34i at 1550 nm.

### Method details

#### Device manufacture techniques

The sub-micron holes were structured via electron beam lithography and etched with silicon ICP technique (Plasmalab system 100 ICP 180). SiO_2_ was deposited with plasma enhanced chemical vapor deposition (PECVD) (PlasmaPro 800 Stratum), the deposition rate is about 50 nm/min. GST and ZnS/SiO_2_ were deposited via sputtering technique (chamber pleasure is 0.5 Pa, voltages are respectively DC 30 W for GST and RF 180 W for ZnS/SiO_2_).

#### Optical measurements

The device was tested with a spectrum system combined with a wide-band picosecond laser, two convex lens and a spectrograph. The laser imposes a signal light on samples. The other lens is behind the sample to lead the light focused in the spectrograph.The ultrafast phase change was induced by a sapphire femtosecond laser with tunable wavelength. Through a Glan-Taylor polarizer, the power of laser beam can be increased or decreased. With micrometer screw, the sample plane can be adjusted.

#### EM characterization

The TEM images is tested via Titan G20years, sample is prepared with FIB etching. To enhance the conductivity, a thin gold film was sprinkled on device surface with evaporation.

### Quantification and statistical analysis

The simulation data is produced by Lumerical FDTD software. Figures shown in the maintext were produced by Origin and Microsoft Visio from the raw data.

### Additional resources

Any additional information about the simulation and data reported in this paper is available from the lead contact on request.

## Data Availability

Any additional information required to reanalyze the data reported in this paper is available from the lead contact upon request.
